# A cross-sectional study describing motivations and barriers to voluntary medical male circumcision in Lesotho

**DOI:** 10.1186/1471-2458-14-1119

**Published:** 2014-10-30

**Authors:** Laura Skolnik, Sharon Tsui, Tigistu Adamu Ashengo, Virgile Kikaya, Mainza Lukobo-Durrell

**Affiliations:** Jhpiego Lesotho, Dolphin House, 23 Motsoene Road, Industrial Area, Maseru, 100 Lesotho; Jhpiego, Baltimore, Maryland USA; Johns Hopkins University Bloomberg School of Public Health, Baltimore, Maryland USA

**Keywords:** Acceptability, Male circumcision, Lesotho, HIV

## Abstract

**Background:**

In February 2012, the Lesotho Ministry of Health launched a national voluntary medical male circumcision (VMMC) program. To assess the motivations for seeking VMMC, a cross-sectional mixed methods study was conducted among clients aged 18 years and older at four sites.

**Methods:**

A total of 161 men participated in individual survey interviews and 35 participated in four focus group discussions.

**Results:**

Men sought medical circumcision for the following main reasons: protection against HIV (73%), protection from other sexually transmitted infections (62%), and improved penile hygiene (47%). Forty percent learned about VMMC through friends who had already accessed services. According to these men, perceived concerns hindering service uptake include fear of pain (57%), a female provider (18%), and “compulsory” HIV testing (15%).

**Conclusions:**

The study provides important insights into the motivations of clients seeking VMMC services. Findings can be used by the national VMMC program to attract more clients and address barriers to uptake.

## Background

Lesotho has the world’s second highest HIV prevalence, estimated at 23% among adults 15–49 years old [1]. As part of Lesotho’s efforts to address the HIV/AIDS crisis, the Lesotho Ministry of Health (MOH) launched a national voluntary medical male circumcision (VMMC) program in February 2012. A coordinated, rapid scale-up of VMMC can avert one HIV infection for every five circumcision performed in Lesotho [2], and it can reduce the transmission of sexually transmitted infections (STIs), such as herpes simplex virus and human papillomavirus, an established precursor to cervical cancer [3, 4].

Prior to the launch of the VMMC program, fewer than 1,000 circumcisions were performed in public hospitals annually [5], although the practice of traditional circumcision and initiation is commonplace, particularly in rural areas [6]. In order to reach 80% VMMC coverage over a five-year period, the Lesotho MOH set a VMMC target of 317,215 men between 15 and 49 years of age, an ambitious target given the traditionally low number of facility-based VMMCs [5]. In the early phase of the program, the MOH decided not to undertake mass communication activities to promote VMMC until services were firmly established in all district hospitals; only posters with information about the availability of VMMC services were displayed in health facilities. Despite the lack of mass communication activities to promote VMMC, once the procedure was available, large numbers of men sought these services at district hospitals. From February 2012 to December 2013, more than 42,000 circumcisions were performed in district hospitals across Lesotho and in a few clinics in Maseru [1].

In this study, we sought to explore the characteristics, motivations, and perceived barriers to seeking VMMC services of Basotho men who were medically circumcised during this initial scale-up phase in order to inform the long-term strategy of the national VMMC program scale-up. We considered these clients to be “early adopters” because they sought VMMC services during the early phase of the VMMC program with minimal demand creation activities on the part of the national program.

## Methods

The study protocol and informed consent process were reviewed and approved by the Lesotho Ministry of Health Research and Ethics Committee (ID 65/2012) and the Johns Hopkins Bloomberg School of Public Health Institutional Review Board (IRB 00004673).

### Study design

This study utilized a cross-sectional design with parallel collection of quantitative and qualitative data. Quantitative data were collected to characterize the clients and their motivations and perceived barriers to seeking VMMC services. Qualitative focus group discussion data were collected in parallel with the quantitative surveys to gain a deeper understanding of the motivations and perceived barriers of VMMC by probing beyond the “what” questions to understand “why” and “how.” Data were collected over six weeks, from May 22 to July 1, 2013. Quantitative data were collected through trained interviewers who administered a structured questionnaire with some open-ended questions. Qualitative focus group discussion (FGD) data were collected through a trained moderator using semi-structured interview guides with open-ended questions and probes. The survey component of the study involved four sites, purposively selected to represent client populations living in different locales: urban (Carewell and Apex Clinics), peri-urban (Ntsekhe Hospital), and rural (Mokhotlong Hospital). Compared to the urban sites, client flow was low at the peri-urban and rural sites and it was difficult to recruit participants at these sites. For this reason, FGDs were conducted only at the two urban sites. Participants for the FGD were organized into two age groups (18 to 26 years and 27 years and older) in order to promote ease of discussion among men of similar ages. Quantitative and qualitative data were analyzed separately and then compared for triangulation—to see if key findings were consistent and supported each other.

### Sampling criteria and sample sizes

Non-random convenience sampling was used to select eligible individuals (males, at least 18 years old, and registered for VMMC at the health facility) to participate in the survey and FGDs. This sampling technique was used because it was not feasible to generate a complete list of clients registered for VMMC services each day to facilitate randomized or systematic sampling of study participants. Men who participated in the survey were not invited to participate in the FGDs. Overall, a total of 161 men participated in a survey interview. Based on prior literature that 50% of men who seek circumcision do so to protect against HIV infection, we determined that 160 men were needed in the study to accurately estimate the proportion of adults who desire circumcision to protect themselves against HIV with 95% confidence [7]. A total of 35 men participated in four FGDs.

### Data collection and management

Upon arrival at the health facility, potential study participants were screened for eligibility and interest in joining the study. Interested persons were taken to a private space—where they could not be seen or heard by health care providers and other clients in the facility—to undergo the informed consent process individually with a trained interviewer. This process included an explanation of how the participant was selected and the risks and benefits of joining the study. Individuals who gave verbal consent were interviewed prior to receiving VMMC services. All interviews (survey and FGD) were conducted in Sesotho, the national language, by trained moderators on the data collection team. No names were recorded for either component of the study and each study participant was assigned a unique identifier to protect his identity and maintain confidentiality. Survey data were double entered into EpiInfo 7.0, compared for inconsistency in data entry, and corrected before export into SAS 9.2 for additional data cleaning, recoding of variables, and statistical analysis. FGDs were audio-taped, transcribed verbatim in Sesotho, reviewed for accuracy, and then translated verbatim into English. The data collection team reviewed the English and Sesotho transcripts together and annotated each English transcript, including narrative examples and Sesotho phrases, to ensure a culturally appropriate interpretation of the text. Sesotho words that had no equivalent translation in English were kept in their original form.

### Data analysis

All statistical analyses were performed using SAS software (SAS Institute, Cary, NC, USA, version 9.2). Descriptive statistics are presented for all variables, overall, and by urban–rural status. Carewell and Apex are grouped together as urban sites and Ntsekhe and Mokhotlong are grouped together as rural sites. Continuous variables were summarized using means, standard deviations [SDs], minima, and maxima. Categorical variables were summarized with frequencies and percentages. Qualitative text were analyzed by the study team [ST, LS, VK, and MLD] using a theme content approach. Each transcript was read and re-read to answer questions on the *a priori* topics set by the study objective, to identify emergent themes, and to develop a codebook. Themes were coded and stored in Atlas.Ti6.0. Coding reports were generated to analyze each theme and findings were summarized in a memo.

## Results

### Study participant characteristics

The socio-demographic characteristics of the 161 survey respondents are summarized in Table 1. About 70% of the survey respondents sampled from the clinics were young men aged 18–25 (data not shown in tables). Greater proportions of younger men, aged 18–25, were never married and did not have a sexual partner in the last three months, compared to older men, 26 years and older (data not shown in tables). The mean age overall was 24.2 years (SD 7.0). Nearly three-quarters of the men were never married (74.5%) and reported living in an urban area (73.9%).

**Table 1:** **Summary of survey participant characteristics**, **by urban**–**rural status and total**

Socio-demographic characteristics	Maseru urban clinics (Carewell, Apex) N = 73	Non-maseru rural clinics (Ntsekhe, Mokhotlong) N = 88	Total N = 161
	n (%)	n (%)	n (%)
Age			
Mean (SD)	26.2 (7.6)	22.6 (6.2)	24.2 (7.0)
Min, Max	18, 48	18, 49	18, 49
Marital status*			
Never married	47 (64.4)	73 (83.0)	120 (74.5)
Married	25 (34.3)	15 (17.1)	40 (24.8)
Cohabitating	1 (1.4)	0 (0.0)	1 (0.6)
Village of residence			
Urban	66 (90.4)	53 (60.2)	119 (73.9)
Rural	7 (9.6)	35 (39.8)	42 (26.1)
Religion			
Roman Catholic	44 (60.3)	36 (40.9)	80 (49.7)
Lesotho Evangelical	6 (8.2)	18 (20.5)	24 (14.9)
Anglican	8 (11.0)	12 (13.6)	20 (12.4)
Pentecostal	9 (12.3)	8 (9.1)	17 (10.6)
Other Christian	2 (2.7)	6 (6.8)	8 (5.0)
Other	2 (2.7)	4 (4.6)	6 (3.7)
None	2 (2.7)	3 (3.4)	5 (3.1)
*Missing*		1 (1.1)	1 (0.6)
Highest education completed			
None	2 (2.7)	1 (1.1)	3 (1.9)
Primary	16 (21.9)	30 (34.1)	46 (28.6)
Secondary	17 (23.3)	25 (28.4)	42 (26.1)
High school	27 (37.0)	26 (29.6)	53 (32.9)
Tertiary	11 (15.1)	6 (6.8)	17 (10.6)
Employment status			
Student	26 (35.6)	49 (55.7)	75 (46.6)
Unemployed	8 (11.0)	15 (17.1)	23 (14.3)
Self-employed	6 (8.2)	4 (4.6)	10 (6.2)
Employed, part-time	7 (9.6)	2 (2.3)	9 (5.6)
Employed, full-time	26 (35.6)	18 (20.5)	44 (27.3)
Number of sexual partners in the past three months			
Mean (SD)	1.2 (1.0)	0.7 (0.8)	0.9 (0.9)
Min, Max	0, 4	0, 3	0, 4
None	19 (26.4)	41 (46.6)	60 (37.3)
1	34 (47.2)	33 (37.5)	67 (41.6)
2	11 (15.3)	12 (13.6)	23 (14.3)
3+	8 (11.1)	2 (2.3)	10 (6.2)
*Missing*	1	0	1 (0.6)

*Proportion in table may not add up to 100.0*% *because of rounding*; *no participants were divorced, *separated*, *or widowed.*

The socio-demographic characteristics of the 35 FGD participants are summarized in Table 2. The age variation observed across sites reflects the purposive sampling strategy of forming groups of younger men aged 18 to 26 years and older men aged 27 years and older. Younger men were interviewed in Carewell 2 and 3 and, as expected, the vast majority of these participants had never married and had completed fewer years of education. In Carewell 1 and Apex, the FGD was conducted with the older men, and a majority of these men were married. Similar to the survey respondents, nearly all of the FGD participants reported residing in an urban area.

**Table 2:** **Summary of focus group discussion participant characteristics**, **by group and total**

Socio-demographic characteristics	Carewell 1 N = 10	Carewell 2 N = 10	Carewell 3 N = 7	Apex N = 8	Total N = 35
	n (%)	n (%)	n (%)	n (%)	n (%)
Age					
Mean (SD)	33.1 (2.8)	20.5 (1.6)	21.9 (3.2)	32.4 (4.1)	27.1 (6.6)
Min, Max	30, 37	18, 23	18, 26	27, 39	18, 39
Marital status*					
Never married	2 (20.0)	9 (90.0)	6 (85.7)	2 (25.0)	19 (54.3)
Married	8 (80.0)	1 (10.0)	1 (14.3)	6 (75.0)	16 (45.7)
Village of residence					
Urban	10 (100.0)	10 (100.0)	5 (71.4)	8 (100.0)	33 (94.3)
Rural	0 (0.0)	0 (0.0)	2 (28.6)	0 (0.0)	2 (5.7)
Highest education completed					
None	0 (0.0)	9 (90.0)	3 (42.9)	0 (0.0)	12 (34.3)
Primary	2 (20.0)	1 (10.0)	2 (28.6)	2 (25.0)	7 (20.0)
Secondary	1 (10.0)	0 (0.0)	0 (0.0)	1 (12.5)	2 (5.7)
High school	0 (0.0)	0 (0.0)	1 (14.3)	3 (37.5)	4 (11.4)
Tertiary	7 (70.0)	0 (0.0)	1 (14.3)	2 (25.0)	10 (28.6)
Employment status					
Unemployed	5 (50.0)	1 (10.0)	1 (14.3)	0 (0.0)	7 (20.0)
Self-employed	3 (30.0)	3 (30.0)	3 (42.9)	1 (12.5)	10 (28.6)
Employed, part-time	2 (20.0)	5 (50.0)	1 (14.3)	1 (12.5)	9 (25.7)
Employed, full-time	0 (0.0)	1 (10.0)	2 (28.6)	6 (75.0)	9 (25.7)

*Proportion in table may not add up to 100.0*% *because of rounding*; *no participants were divorced, *separated*, *or widowed.*

### How study participants learned about VMMC

Responses among survey participants regarding how they learned about VMMC are summarized in Table 3. Overall, 40% of the respondents learned about male circumcision through friends who had already accessed it, 7% through friends who had not accessed it, and 15% from family members. The quantitative findings are consistent with the qualitative data. In addition, the qualitative data suggest that the MOH is an influential opinion leader on men’s uptake of medical circumcision services.

**Table 3:** **How survey participants learned about VMMC**, **by urban**–**rural status and total**

How you learned about circumcision in facility:	Maseru urban clinics (Carewell, Apex) N = 73	Non-maseru rural clinics (Ntsekhe, Mokhotlong) N = 88	Total N = 161
	n (%)	n (%)	n (%)
Family members	6 (8.2)	18 (20.5)	24 (14.9)
Friends who accessed VMMC	38 (52.1)	27 (30.7)	65 (40.4)
Friends who have not accessed VMMC	8 (11.0)	3 (3.4)	11 (6.8)
Health booklet	0 (0.0)	7 (8.0)	7 (4.4)
Radio	8 (11.0)	10 (11.4)	18 (11.2)
TV	7 (9.6)	0 (0.0)	7 (4.4)
Mobilization at school	1 (1.4)	16 (18.2)	17 (10.6)
Mobilization other than at school	2 (2.7)	4 (4.6)	6 (3.7)
Other: Carewell referral, health facility visit, hospital gate poster	3 (4.1)	3 (3.4)	6 (3.7)

*Open*-*ended questions where participant may give none*, *one*, *or more than one response therefore proportion in table may not add up to 100.0%.*

The most commonly cited way a survey participant learned about medical circumcision was from friends: at Carewell (~67%), Apex (~46%), and Mokhotlong (~38%), and it was the second most commonly cited way at Ntsekhe (~26%). The FGD participants expanded on this theme and reported experiencing peer pressure from friends who encouraged them to get circumcised because they believed a circumcised penis had a better shape, that male circumcision would enhance sexual pleasure, and that women preferred having sex with a circumcised man.

In addition to friends, family members such as mothers, fathers, grandparents, siblings, and children were influential in the survey respondents’ decision to get circumcised. Approximately 6% of participants reported learning about the importance of male circumcision from female family members. The vast majority (~91%) of survey respondents perceived family approval of male circumcision. The top reasons cited for family approval among all the survey respondents were health related—to reduce HIV risk (~30%), reduce STIs (~11%), improve hygiene (~9%), and to promote health in general (~6%). Sexual partners, including girlfriends or wives, were also an important influence on men’s decision to seek male circumcision. Many FGD participants said their sexual partners explicitly encouraged them to get circumcised, and nearly all the survey respondents (~98%) who had a sexual partner in the past three months of the date of interview, believed their sexual partner approved of male circumcision. However, only ~58% said their sexual partners knew they were getting circumcised.

Beyond the men’s interpersonal social networks, the Lesotho MOH was seen as a trusted opinion leader and source of information on male circumcision. Eleven percent of survey respondents said they learned about male circumcision through MOH radio awareness-raising health talks.

### Factors motivating men to seek VMMC

Factors motiving men to seek VMMC include: disease prevention, hygiene, sexual pleasure, and social recognition for circumcised men. As summarized in Table 4, the leading reasons for circumcision among all survey respondents are self-protection against HIV (~73%), self-protection against STIs (~62%), promotion of penile hygiene (~47%), improving sexual pleasure for self (~9%), improving sexual pleasure for partner (~8%), and gaining social prestige (5%).

**Table 4:** **Motivations for seeking VMMC services among survey participants**, **by urban**–**rural status and total**

Reasons for seeking VMMC	Maseru urban clinics (Carewell, Apex) N = 73	Non-maseru rural clinics (Ntsekhe, Mokhotlong) N = 88	Total N = 161
	n (%)	n (%)	n (%)
Protect self against HIV	52 (71.2)	66 (75.0)	118 (73.3)
Protect self against STIs	48 (65.8)	51 (56.0)	99 (61.5)
Penile hygiene	36 (49.3)	39 (44.3)	75 (46.6)
Improved sexual pleasure for self	8 (11.0)	6 (6.8)	14 (8.7)
Improved sexual pleasure for partner	6 (8.2)	7 (8.0)	13 (8.1)
Prestige	5 (6.9)	3 (3.4)	8 (5.0)
Protect partner against STIs	4 (5.5)	3 (3.4)	7 (4.4)
Protect partner against HIV	3 (4.1)	4 (4.6)	7 (4.4)
Medical reason	2 (2.7)	2 (2.3)	4 (2.5)
Other: cultural belief, easily accessible, spouse, peer pressure, “don’t know”	9 (12.3)	3 (3.4)	12 (7.5)

*Open*-*ended questions where participant may give none*, *one*, *or more than one response therefore proportion in table may not add up to 100.0%.*

The qualitative findings on why men sought male circumcision services were consistent with the quantitative survey results. The most salient reasons were to promote health, for example, to reduce the risks of contracting HIV and STIs. “It is so one can live healthy even if one finds oneself in the way of diseases, they do not infect you with great intensity or create problems for you.” (Older man from Apex) In addition to disease prevention, men wanted the foreskin removed to improve penile cleanliness. “I think our expectations are maybe to get rid of the filth that usually hides under the skin.” (Young man from Carewell) Another salient reason was to enhance sexual attractiveness, performance, and pleasure. “We are also doing it for sex. Indeed, we have been told that when the skin has been removed, you become number one…. We want her to always say ‘this guy has something nice.’ Ehe.” (Young man from Carewell).

Some of the FGD participants explained that male circumcision was an influential cultural practice for establishing masculinity; they sought circumcision to be a “real” or “complete” man. “Actually, you will find that when your friends see you that way [not circumcised] you will actually find that you do not feel like a real man. It does not allow you to speak with other men in a confident manner.” (Older man from Apex) While some FGD participants believed medical circumcision can enhance one’s prestige in the community, others did not think medical circumcision achieved the same prestige and social recognition as circumcision performed during a traditional initiation. For example, a participant said, “I am a Mosotho [singular term for man from Lesotho], but I am not excited about the idea of removing the skin in the mountain [traditional circumcision] so in my head I am still a man even when I have done this one [hospital circumcision]” (young man from Carewell). However, another participant countered, “Honestly, most people where I live have done it in the mountain. For them, there is only one way of doing this, which means they despise it [hospital circumcision] greatly. When you go there, it’s like you are still a woman. They say things like that because they do not understand that we see things differently, and that is what makes life with them painful. They sideline us even in small conversations… they do things like that.” (Young man from Carewell).

### Perceived barriers to VMMC

While survey and FGD participants perceived multiple benefits to medical circumcision and were motivated to seek VMMC services, many individuals also expressed concerns that have delayed them or prevented their friends from seeking medical circumcision. The salient barriers to VMMC across both survey and FGD data were fears of pain, compulsory HIV testing, cost, and being attended by female health staff.

### Fear of pain

Some men feared intense pain because they saw circumcision as an invasive procedure. “Honestly, I think it is very painful because even if you pinch yourself a little bit with a zip, you will really feel pain. How much more when it has been cut completely? So this is another thing that scares me when I have to do it”. (Young man from Carewell) Men’s fear of pain was not limited to cutting off the foreskin—pain was associated with the entire process of circumcision: waiting for the procedure and observing men who have just been circumcised, pain from local anesthetic injections, pain from having stitches removed, and pain through the healing period, particularly when one has unintended erections. Fear of erections as a cause of pain in the weeks following circumcision was emphasized in all four FGDs, and some participants even requested a medication to prevent erections. “I was hoping that maybe we will be given some pills … so that it [the penis] sleeps or so that it does not cause any trouble [pain from erection].” (Young man from Carewell).

Nearly 60% of the survey participants reasoned that it is the fear of pain that has prevented other men from seeking VMMC (Table 5). Many participants believed that time off from school or work is needed to recover from the pain of circumcision, and a perceived lack of time (~20% lack time off from school and ~17% lack time off work, Table 5) has caused men to delay seeking VMMC. “Actually there are many people who are still afraid that if it is painful he will not be able to go to work. They do not have time.” (Older man from Carewell).

**Table 5:** **Perceived reasons preventing or delaying men**’**s access to VMMC**, **by urban**–**rural status and total among survey participants**

Perceptions	Maseru urban clinics (Carewell, Apex) N = 73	Non-maseru rural clinics (Ntsekhe, Mokhotlong) N = 88	Total N = 161
	n (%)	n (%)	n (%)
Perceived reasons preventing men from accessing VMMC			
Long wait time	4 (5.5)	1 (1.1)	5 (3.1)
Mixing young and old clients	2 (2.7)	3 (3.4)	5 (3.1)
Being attended to by female staff	7 (9.6)	22 (25.0)	29 (18.0)
HIV testing	15 (20.6)	9 (10.2)	24 (14.9)
Fear of pain	45 (61.6)	46 (52.3)	91 (56.5)
Fear of injection	4 (5.5)	5 (5.7)	9 (5.6)
Long healing time, abstinence	3 (4.1)	4 (4.6)	7 (4.4)
Concern about VMMC safety	3 (4.1)	2 (2.3)	5 (3.1)
Preference for traditional male circumcision.	2 (2.7)	5 (5.7)	7 (4.4)
Other: lack transport fare, VMMC doesn’t give full HIV protection, don’t know where to get VMMC, poor service, bad attitude from health staff	8 (11.0)	7 (8.0)	15 (9.3)

*Open*-*ended questions where participant may give none*, *one or more than one response therefore proportion in table may not add up to 100.0%.*

### Fear of HIV testing and knowing one’s serostatus

Fear of HIV testing and learning one’s serostatus was another factor perceived to prevent men from seeking VMMC. HIV counseling and testing (HCT) is offered to every client seeking VMMC; HCT is performed after group education on the risks and benefits of medical circumcision and individual counseling with the nurse. A substantial proportion of survey participants (~15%, Table 5) and FGD participants perceived HIV testing to be compulsory for the VMMC program (although it is, in fact, not compulsory). These study participants believed that other men were not seeking medical circumcision in order to avoid an HIV test. “Some people do not come here for the simple reason that they actually feel like they are being forced to test. Actually, you would not receive the services unless you test and know your status. And so it put some people in a difficult position, Ntate.” (Young man from Carewell) Some men did not want the HIV test because they were afraid of finding out their serostatus. “Testing to know your status, I mean, it is scary on its own. That is the one thing that makes one to stress and think twice before coming here, but I guess it is a process that you have to go through to get to what you need [circumcision]. I mean, you stress and think twice before coming here. Even now, I have not made a final decision in my head.” (Young man from Carewell).

### Cost

Cost, particularly indirect costs for transport, was another barrier to VMMC. “We come from distant places to get services. Sometimes, some of us would be willing to go but could not because of [a] lack of transport money.” (Younger man from Carewell) Notably, participants felt burdened by the cumulative transport costs associated with VMMC because they were required to make multiple visits to the clinic to register for the service, get circumcised, and return for several follow-up visits.

### Female health staff

Nearly 20% of the survey participants believed that having a female medical professional perform the circumcision prevented other men from seeking medical circumcision (Table 5). A FGD participant explained that Basotho men feel shy when unclothed in front of women. “People who operate [on] us are predominantly women. I think as Basotho children we still have a problem of feeling free in the presence of women. So I suggest they increase the number of men working here. That way we would feel free to ask anything and do anything.” (Older man from Carewell).

## Discussion

### Characterizing men who seek VMMC in Lesotho

The study population is a sample of adult men who sought out medical circumcision in the early phase of the country’s national scale-up of VMMC when there were few if any mass communication activities to promote medical circumcision. They are largely young (~70% between ages 18–25), never married (~75%), and reside in an urban area (~74%). The demographic characteristics of the adopters of VMMC in our study are quite different in comparison to men who have been circumcised in the general population. According to the Lesotho 2009 Demographic and Health Survey, the vast majority of circumcised men aged 15–49 at the time of the interview reported being circumcised at a younger age, between 13–19 years (54–93%), and a majority of them (~59%) resided in a rural locale [8]. Differences in age are expected for two reasons: first, our study was only able to include men at least 18 years of age so we are unable to collect information from younger VMMC clients, and second, men who reported being circumcised prior to the time of the national VMMC roll-out were likely to have been circumcised through traditional initiation, which is carried out when boys have come of age, which is in early adolescence (~13 years). The large representation of VMMC clients from urban areas is not surprising because VMMC services were first piloted and scaled-up in Maseru before services were incrementally decentralized to district hospitals outside of the capital.

### Facilitators: protective effect against diseases, hygiene, sexual pleasure, and peers

Health was the main reason why men sought VMMC. Most men cited reduced risks of contracting HIV and STIs as reasons they came for circumcision. Many men expressed a sense of individual responsibility to reduce one’s risk of acquiring HIV and STIs and a communal responsibility to reduce the rates of HIV in Lesotho. This finding is not surprising because HIV is perceived to be a pervasive and serious problem in Lesotho. HIV prevalence is high—nearly one out of four adults aged 15–49 is infected with HIV—and HIV awareness is near universal—95% and 97% of men and women, respectively, report that they have heard of HIV [9]. The belief in the partial protective effect of male circumcision against HIV infection and other STI is also a major motivating factor for VMMC in other African countries where there is high HIV prevalence, such as Kenya [10], Botswana [11], Malawi [12], Zambia [13], Zimbabwe [14], and South Africa. This finding suggests that medical circumcision to prevent HIV and STIs remains a salient, acceptable, and effective message to promote the uptake of VMMC in Lesotho and other countries with a high burden of HIV.

Circumcision to improve penile hygiene is another motivating factor for VMMC among Basotho men. This theme is also consistent and in common with findings in acceptability studies in Kenya [10], Malawi [12], Zambia [13], Zimbabwe [14], and Tanzania [15]. According to these studies, men believed the foreskin harbors dirt, that with removal of the foreskin, the penis will be easier to bathe and keep dry [10, 13, 16].

Sex was another factor driving men to seek VMMC. Specifically, men sought medical circumcision to enhance their sexual attractiveness and performance. There was a pervasive perception among both younger and older men interviewed in the FGDs that women preferred men who were circumcised. Many men believed that they and their sexual partners would experience increased pleasure through increased sensitivity in the head of the penis or by decreased sensitivity, which would allow for delayed ejaculation. Similar findings are also reported in two studies in South Africa [17, 18] as well as studies in Malawi [12] and Zambia [13]. In contrast, a study conducted among men from Nyanza Province, Kenya, found that men there believed circumcision to reduce penile sensitivity, resulting in a loss of sexual desire and pleasure [10]. Although circumcision has not been found to affect sexual performance or satisfaction [19], it is interesting that this belief continues to surface among men in Lesotho and other countries in the region.

The role of peers was inextricably intertwined with motivating factors affecting men’s adoption of medical circumcision, perhaps because our study was composed largely of young men (~70% less than 26 years). Participants typically discussed issues of penile hygiene and sexual pleasure within the context of conversations with friends. During these recollections, participants expressed feelings of social pressure to be medically circumcised. Common expressions include, “My friends are really encouraging me” and “I am the only one remaining.” This finding is consistent with a recent study on the acceptability of male circumcision in Kenya a year after the launch of the national male circumcision program, which found social pressure to be a major facilitator of uptake among young men [20]. Several FGD participants compared not being circumcised to instances in which one may be socially ridiculed, such as being a “bald-headed” man. Using mockery as a form of peer pressure for circumcision was also observed in Tanzania where men who were not circumcised were belittled for being “dirty” [16]. Mockery may conform to traditional ways men use to cajole each other to be circumcised. For example, a qualitative study on male circumcision in Uganda found that ridicule and stigmatization of uncircumcised males is common in traditional circumcising areas because young men use circumcision as a means to gain social acceptance [21].

### Barriers: pain, cost, and HIV testing

This study focused on those men who came for services, and by design, this study cannot directly answer questions about why some men refuse medical circumcision. However, the study participants’ perceptions of barriers to accessing VMMC can offer important insight on why some men delay or refuse to seek VMMC. The key barriers that emerged from both the quantitative and qualitative data were: fear of pain, cost, and fear of HIV testing.

Fear of pain during and following the procedure was the most common perceived barrier to VMMC in this study and is also universally cited as an obstacle to VMMC in acceptability studies conducted in Kenya, Botswana, Malawi, Zambia, Zimbabwe, and South Africa [11–15, 17, 18, 22–24]. It is noteworthy that participants were interviewed prior to circumcision so they were speaking about *perceived* pain, not pain based on experience. Participants’ perception of pain likely derives from a combination of observations and rumors: observing recently circumcised men walk with a shuffling gait and conflating their observations with rumors of pain spread among peers and those waiting for VMMC services. This finding on apprehension of pain as a result of rumors and stories has also been noted by Westercamp and Bailey [23]. Addressing fear of pain is critical as it is one of the main barriers of seeking VMMC. This is an important area for programmatic development because most communication strategies to date only stress that medical circumcision works to reduce men’s risk for HIV infection and has the additional benefits of improving hygiene and reducing cancer risk.

Lack of funds for transport was another barrier to VMMC in Lesotho, and is also a barrier to access in Kenya, Malawi, and Zambia [12–14, 22]. This finding is congruent with a health economics study, which found that a VMMC client potentially spends 22% of the annual $75 out-of-pocket expenditure that a typical Basotho spends on health just for transportation to access circumcision services (assuming he makes the two post circumcision follow-up visits that are part of the three visit VMMC service protocol) [25]. This expense occurs even with efforts to provide the service and any medications free of charge. For this reason, participants recommended even more decentralized services (from district hospitals to primary health care centers) so that men could access services closer to home, and some even suggested a same-day service to eliminate at least one extra trip to the clinic.

Fear of HIV testing and knowing one’s serostatus is another perceived barrier to VMMC in Lesotho. Men’s reluctance to get HIV tested in VMMC services is also observed in Lesotho’s general HCT program. The rate of HIV testing and receiving results is very low; less than 50% of men under 40 years have *ever* been tested for HIV and received results [9], and the proportion of men aged 15–49 who have been tested and received results *in the last 12 months* is even smaller, at 24% [26]. Lesotho’s low rate of HIV testing among men is similar to other countries in Eastern and Southern Africa [26], including Kenya and Zimbabwe where male uptake of HIV testing is commensurate with Lesotho. However, this is the first study to document the fear of HIV testing as a key barrier to VMMC. Research suggests that marital status is an important correlate on the uptake of HCT. Being married is a key mechanism for getting men tested for HIV because of premarital HCT or the institutionalization of HCT for couples as part of antenatal care [27, 28]. It is possible that this study population is unique from other studies because the men are mainly young and never married.

### Limitations

The study findings have limited generalizability to the larger population in Lesotho because study participants were conveniently sampled among four non-randomly selected sites. Also, the representativeness of study findings among VMMC clients cannot be established because screening data were not collected on all the VMMC clients. Another limitation is the lack of data variability in demographic variables, such as age (~80% less than 26 years), marital status (~75% never married), or urban/rural residence (~75% urban), which has limited our ability to make comparisons and test for statistical differences across groups.

## Conclusions

This study provides important insights into the motivations and challenges of clients seeking medical circumcision services in the early phase of the national implementation of VMMC in Lesotho. The findings from both quantitative and qualitative data sources suggest that circumcision for health, especially HIV and STI prevention, is the predominant motivator for VMMC, and this continues to be an effective message for promoting medical circumcision in settings where there is a high burden of HIV. There are considerable barriers to circumcision, including fear of pain, fear of HIV testing, and indirect costs associated with VMMC. However, these barriers are not insurmountable as, evidently, study participants were able to overcome them and ultimately seek medical circumcision. These findings can inform the national VMMC program, which can build on what we have learned in this study and develop mass communication messages that specifically addresses the fears and concerns experienced by men who sought VMMC. However, additional research to understand the impact of fear of HIV testing on VMMC uptake in the southern Africa region is needed.

## Abbreviations

FGD: Focus group discussion
HCT: HIV counseling and testing
MOH: Ministry of Health
SD: Standard deviation
STI: Sexually transmitted infection
VMMC: Voluntary medical male circumcision.
